# Targeting the DNA damage response and repair in cancer through nucleotide metabolism

**DOI:** 10.1002/1878-0261.13227

**Published:** 2022-05-28

**Authors:** Thomas Helleday, Sean G. Rudd

**Affiliations:** ^1^ Science for Life Laboratory Department of Oncology‐Pathology Karolinska Institutet Stockholm Sweden; ^2^ Department of Oncology and Metabolism, Weston Park Cancer Centre University of Sheffield UK

**Keywords:** cancer, DNA damage response, dNTP metabolism, MTH1, MTHFD2, SAMHD1

## Abstract

The exploitation of the DNA damage response and DNA repair proficiency of cancer cells is an important anticancer strategy. The replication and repair of DNA are dependent upon the supply of deoxynucleoside triphosphate (dNTP) building blocks, which are produced and maintained by nucleotide metabolic pathways. Enzymes within these pathways can be promising targets to selectively induce toxic DNA lesions in cancer cells. These same pathways also activate antimetabolites, an important group of chemotherapies that disrupt both nucleotide and DNA metabolism to induce DNA damage in cancer cells. Thus, dNTP metabolic enzymes can also be targeted to refine the use of these chemotherapeutics, many of which remain standard of care in common cancers. In this review article, we will discuss both these approaches exemplified by the enzymes MTH1, MTHFD2 and SAMHD1. © 2022 The Authors. Molecular Oncology published by John Wiley & Sons Ltd on behalf of Federation of European Biochemical Societies.

## Graphical abstract

The exploitation of the DNA damage response and DNA repair proficiency of cancer cells is an important anticancer strategy. The replication and repair of the DNA molecule are dependent upon the supply of deoxynucleoside triphosphate (dNTP) building blocks, which are produced and maintained by nucleotide metabolic pathways. Here, we will discuss how targeting these pathways can be a promising anticancer strategy.

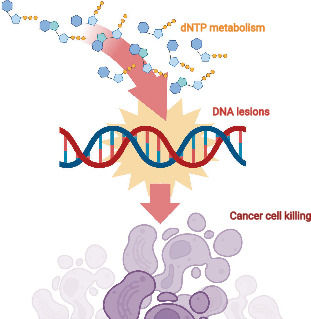

**Abbreviations**
AGS: Aicardi‐Goutières syndrome
ALL: acute lymphoblastic leukaemia
AML: acute myeloid leukaemia
and Vpx: viral protein‐X
Ara‐C: cytarabine
AS1: allosteric site 1
AS2: allosteric site 2
DDR: DNA damage response
dF‐dC: gemcitabine
dNTP: deoxynucleoside triphosphate
FDA: United States Food and Drug Administration
HU: hydroxyurea
MTH1: human MutT homologue 1
MTHFD2: methylenetetrahydrofolate dehydrogenase/cyclohydrolase 2
OGG1: 8‐oxoguanine DNA glycosylase
PNP: purine nucleoside phosphorylase
RNR: ribonucleotide reductase
ROS: reactive oxygen species
SAMHD1: sterile alpha motif (SAM) and histidine–aspartic acid (HD) domain‐containing protein‐1
SHMT1: serine hydroxymethyltransferase 1

## 1 Introduction

All dividing cells require nucleotide building blocks (deoxynucleoside triphosphates, dNTPs) to copy their DNA in the S phase of the cell cycle, to accurately pass on an intact genome to the next generation. Uncontrolled growth signals provided by oncogenes contribute to cancer development by causing replication stress [1, 2], which is when DNA synthesis slows or stalls leading to exposed stretches of excess single‐stranded DNA. This can be caused through a number of distinct mechanisms, including unscheduled DNA synthesis and perturbation of dNTP metabolism [3], which in turn can contribute to genome instability in cancer [4]. Defects in DNA repair pathways can further fuel cancer development through the acquisition of mutations and gene rearrangements [5].

Targeting DNA replication has been an early strategy in the treatment of cancer [6], either through the use of DNA‐damaging agents, such as nitrogen mustards and topoisomerase poisons, which disrupt DNA replication [7], or alternatively through the use of antimetabolites, which can disrupt both DNA and nucleotide metabolism [8]. More recently, a refinement of this approach has been to inhibit the DNA damage response (DDR) as a strategy to overload the cancer cell with cancer‐specific cytotoxic DNA damage, either alone or in combination with classical DNA‐damaging agents. This strategy can selectively target the cancer using, for instance, the synthetic lethal approach exemplified by PARP inhibitors in BRCA‐mutated cancers [9, 10]. One extension of this approach is to target the DDR, together with DNA repair, through inhibiting nucleotide metabolism in cancer to induce cancer‐specific DNA damage [11] or exploit cancer‐specific nucleotide metabolic pathways [12, 13]. In addition, nucleotide metabolism can be targeted to modulate the efficacy of antimetabolite therapies [14, 15], to refine the use of these classical chemotherapies, which is important considering these drugs remain standard of care for many common cancers. In this review, we will discuss both approaches, exemplified by our recent work on the (nucleotide) metabolic enzymes MTH1 (human MutT homologue 1), MTHFD2 (methylenetetrahydrofolate dehydrogenase/cyclohydrolase 2) and SAMHD1 (sterile alpha motif and histidine‐aspartic acid domain‐containing protein‐1).

## 2 MTH1

Many cancers are characterised by high levels of reactive oxygen species (ROS) [16] as a potential consequence of lost redox balance. High levels of ROS can cause oxidative damage to DNA and proteins, leading to mutations or apoptosis, eventually becoming lethal for the cell [17]. High ROS levels are a potential explanation for antioxidant defences being generally upregulated in cancer, and thus, targeting these high ROS levels is emerging as an anticancer strategy [18].

One of the proteins involved in response to oxidative stress is MTH1 (human MutT homologue 1, NUDT1). This enzyme hydrolyses oxidised nucleotides, such as 8‐oxo‐dGTP and 2‐OH‐dATP, in the dNTP pool to prevent the incorporation of the damaged nucleobase into DNA, which can cause a mutation [19, 20]. Early on, it was demonstrated that lung cancer development in *Ogg1*−/− mice, lacking the 8‐oxoguanine glycosylase OGG1, was dependent on a functional MTH1 protein, as *Ogg1*−/− *Mth1−/−* double knockout mice were spared from cancer [21]. This showed validation of MTH1 as an anticancer target in an animal model. The first mechanistic suggestion that MTH1 could be a potential anticancer target was by P. Rai in R. Weinberg’s laboratory, demonstrating that MTH1 was required to prevent the onset of senescence in cancer cells [22]. Following this, ours and other laboratories developed MTH1 inhibitors demonstrating potent anticancer activity [11, 23], generating a broad interest in this protein as an anticancer target, which has resulted in several series of MTH1 inhibitors with differing abilities to kill cancer cells (discussed further in Section 2.3).

### 2.1 Biological roles of MTH1

As the name Human MutT homologue 1 (MTH1) indicates, the protein was first identified in *E. coli* as the product of a mutator gene, and then later cloned and identified to be a dNTPase hydrolysing 8‐oxo‐dGTP to prevent the incorporation of this modified nucleotide into DNA and subsequent mutations [24, 25]. Like the bacterial MutT, the human MTH1 protein also hydrolyses oxidised nucleotides such as 8‐oxo‐dGTP and 2‐OH‐dATP. As loss of MutT in *E. coli* is one of the most mutagenic events in this organism, increasing mutation rates 1000‐fold, it was surprising to observe no increasing mutation rates in *Mth1*−/− mice [26]. This could potentially be explained by putative backup proteins within the NUDIX hydrolase family, for example MTH2 (NUDT15) [27], NUDT5 [28] and MTH3 (NUDT18) [29], which may reduce the burden of oxidised dNTPs. However, the role of NUDT15 [30], NUDT5 [31], NUDT18 [30] and other NUDIX enzymes [32], in sanitation of oxidised dNTP pools in cells has been challenged [20]. Instead, the reason for *Mth1−/−* mice not having an increase in mutation rates is likely explained by the overall low levels of oxidative stress in mammals. Under conditions where external oxidative stress is added, *Mth1−/−* mice are highly sensitive [33]. Outside of oxidised purines, MTH1 also removes methylated purine triphosphates, such as O6‐methyl‐dGTP and N6‐methyl‐dATP, from the nucleotide pool [34, 35].

The expression from the *NUDT1* gene (encoding the MTH1 protein) and cellular 8‐oxo‐dGTPase activity is highly upregulated following induction of ROS by, for instance, ionising radiation (IR) [36] or environmental pollutants [37, 38]. In human cells, the overall ROS levels are described as low, surprisingly even in cancer cells, and are only upregulated during prolonged perturbations such as arrest in mitosis [39, 40]. In mitosis, emerging data indicate the MTH1 protein is important for microtubule polymerisation and binds tubulin directly, together with other tubulin‐controlling GTPases [41]. It makes sense that MTH1 is upregulated together with ROS in mitosis, but the function of MTH1 under such stressed conditions remains unexplored. The biological role of MTH1 binding to mitotic proteins is also currently unclear.

While the loss of MTH1 function in mammalian cells shows surprisingly little phenotype, the overexpression of MTH1 efficiently reduces mutations in mismatch repair (MMR) defective cells [42], reduces risk of Huntington’s disease‐like impairment [43] and increases life expectancy in mice [44].

### 2.2 MTH1 in inflammation and cancer

Oxidative DNA damage and MTH1 are relevant in numerous diseases and disorders, including neurological diseases, which are reviewed elsewhere [45]. Early on, it was reported that MTH1 protein levels are potently upregulated in phytohaemagglutinin‐activated T lymphocytes [46]. This is unsurprising as activated T cells are known to have increased ROS levels, which are related to the glycolytic switch in activated T cells [47], resembling the same glycolytic switch in cancer [48]. Interestingly, a subset of activated T cells show high level of MTH1 [49] and another subset of activated T cells do not show upregulation of MTH1 [50]. MTH1 inhibitor TH1579 efficiently introduced oxidative DNA damage and kills off activated MTH1^high^ T cells at low nm concentrations but is not toxic to resting or activated MTH1^low^ T cells [50]. A therapeutic effect in a murine model of autoimmune hepatitis [49] and experimental autoimmune encephalomyelitis [50] is reported, but the detailed use of MTH1 inhibitors in inflammatory diseases is yet to be established.

The MTH1 protein is reported to be highly overexpressed in many cancers [51, 52, 53], also correlating with an increased 8‐oxo‐dGTPase activity in cancer [54, 55]. It is unclear why MTH1 protein levels and activity are increased, but it is likely related to dysregulated redox balance that causes ROS and MTH1 transcriptional upregulation. Also, as MTH1 activity prevents cancer cells entering ROS‐induced senescence [22, 56, 57], this may be a way for cancer cells to survive.

### 2.3 Clinical MTH1 inhibitor – mechanism of action

**Fig. 1:**
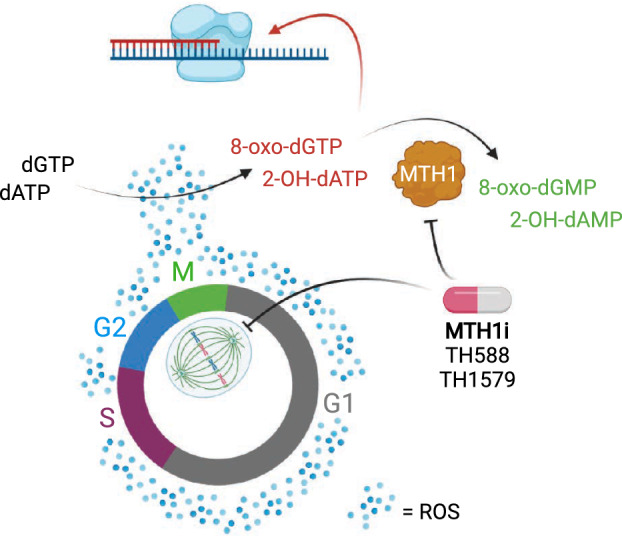
Mechanism of action of clinical MTH1 inhibitors. Schematic detailing the current model for the mechanism of action of MTH1 inhibitors under clinical investigation. TH1579 (karonudib, OXC‐101) and related compound TH588 are dual inhibitors of the catalytic activity of MTH1 and microtubule polymerisation. These compounds arrest cells in mitosis leading to the accumulation of reactive oxygen species (ROS) that subsequently oxidise the purine triphosphate pool. The dNTPase activity of MTH1 is important during this arrest to prevent the use of these modified nucleotides in mitotic DNA synthesis, which is cytotoxic to cancer cells by a yet to be determined mechanism. Figure adapted from [41] and [59], and created in BioRender. [Colour figure can be viewed at wileyonlinelibrary.com]

The MTH1 inhibitor TH1579 (karonudib, OXC‐101) [58] is currently in clinical trials for the treatment of solid (NCT03036228) and haematological cancers (NCT04077307). The model for the mechanism of action of how TH588 (first generation MTH1 inhibitor) and TH1579 (an optimised analogue) kill cancer cells is now fairly well established (Fig. 1). These compounds have dual activities that contribute to their potent antitumour effects, targeting both the catalytic activity of MTH1 and the polymerisation of microtubules. Treatment of cultured cells with these compounds stops them in mitosis and activates the spindle assembly checkpoint (SAC), causing accumulation of ROS. High ROS subsequently oxidises the dGTP pool to generate excess 8‐oxo‐dGTP that can then be incorporated into DNA during mitotic replication, which kills cancer cells [41, 59], although the exact mechanism remains unclear. Several lines of evidence support this model; for example, inhibition of the SAC (either with reversin or with MAD2 siRNA) generates resistance to these MTH1 inhibitors, as this prevents mitotic arrest and the build‐up of excess ROS [41]. Also, replacement of one of the main DNA replicases, DNA polymerase δ (Pol δ), with an error‐prone variant in cells, increases TH588‐induced genomic 8‐oxo‐dG together with mitotic delay and mitotic cell death, linking these phenotypes to DNA synthesis [59]. The activity of TH588 and TH1579 directly on microtubule polymerisation has been characterised *in vitro* [41, 60, 61], and a cocrystal structure of TH588 together with the α/β‐tubulin heterodimer has been resolved, suggesting TH588 occupyies the colchicine site of the GTPase β‐tubulin [61]. Cells expressing a β‐tubulin mutant within the drug‐binding pocket (the TUBB L240F mutant) became more resistant [61], supporting that direct activity of these compounds on β‐tubulin is important for anticancer effect. Alternatively, this mutation could also potentially affect tubulin binding by MTH1. These dual‐function molecules exploit a specific vulnerability of cancer cells to mitotic arrest coupled with loss of MTH1 activity. Separation of these activities, using mitotic poisons to arrest cells in mitosis with cotreatment with a molecule specifically targeting the 8‐oxo‐dGTPase activity of MTH1, synergistically kills cancer cells [41], further supporting the proposed model. These MTH1 inhibitors have a broad anticancer activity in *in vitro* and *in vivo* models [62, 63, 64, 65, 66, 67, 68], and it is interesting to note that while these MTH1 inhibitors are a highly effective anticancer treatment, they are also highly tolerable.

The big discussion is, however, not about the mechanism of action of the TH series inhibitors but if targeting MTH1 contributes to the cell killing observed with these drugs. The reason for this discussion is largely that CRISPR‐Cas9 knockout (KO) of MTH1 is generally well tolerated in cancer cells, and at the same time, these cells are often also sensitive to treatment with TH588 or TH1579. That loss of MTH1 (by siRNA) is tolerable in most cancer cell lines was already reported in our original study in 2014 [11]. Hence, protein loss appears not to recapitulate the effects of the inhibitors, which can be ascribed to edge‐specific genetic (edgetic)‐like perturbation effects, which are very common in the DDR field. Edgetic perturbations were originally described in the context of genetic alterations, such as single amino acid substitutions, which would result in partially functional gene products with altered biochemical and biophysical interaction(s), so‐called as these perturb the edges of interactome network models [69]. Along similar lines, the inactivation of an enzymes catalytic activity with a small molecule, akin to a point mutation ablating activity, could also have other effects upon the biochemical and biophysical properties of this enzyme in cells. Notably, some MTH1 CRISPR‐Cas9 KO cells show a similar level of resistance to TH588 as the TUBB L240F mutant [70], supporting that MTH1 mediates the toxic effects of TH588. Also, structurally distinct MTH1 inhibitors that do not interfere with tubulin polymerisation *in vitro* demonstrate a similar mitotic arrest as TH1579 and TH588 [41], likely interfering with the role of MTH1 in mitosis. Furthermore, some potent MTH1 inhibitors fail to break the protein–protein interaction between MTH1 and α‐tubulin, while others do [41], demonstrating that inhibitors have distinct effects from protein loss, and are not just simple enzyme activity inhibitors. Clearly, edgetic‐like perturbation effects are caused by the MTH1 inhibitors that go beyond simply inhibiting the enzyme and that are of importance for the cytotoxicity of the compounds. Also, we argue these MTH1 inhibitors are not to be confused with microtubule poisons, as they: (a) largely show distinct mitotic defects different from microtubule poisons, (b) effectively target microtubule‐resistant cancers (unpublished), (c) are highly tolerable and not toxic to nontransformed cells and (d) introduce oxidative DNA damage. It will be interesting to follow the clinical development of TH1579.

We call upon more research efforts to shed light on the complex role of the MTH1 protein, particularly interactions in mitosis, together with a more detailed evaluation of TH588 and TH1579 that takes into account edgetic‐like perturbation effects. As the compounds show potent anticancer activity without being generally toxic, in‐depth detail of the exact mechanisms may give further insights to unravel new cancer biology that can be targeted.

## 3 MTHFD2

The DDR needs not only to signal the damage and attract DNA repair enzymes, but it also needs to supply dNTPs to complete the repair. Cancer cells rely on a different set of metabolism enzymes from normal cells owing to the Warburg effect. Previously, we validated the glycolytic PFKFB3 enzyme, preferentially expressed in cancer, as an anticancer target and described the small molecule KAN0438757 that efficiently blocked repair synthesis by depletion of the local dNTP pool at sites of DNA damage [12]. The differential use of metabolism proteins has also generated a lot of interest in the serine and glycine pathways as major drivers of rapid cell proliferation, an effect largely mediated by the folate/one‐carbon metabolism pathway [71, 72, 73]. A particular interest has been generated in the MTHFD2 enzyme as it is oncofetal, being expressed during early embryogenesis, silenced in adult cells and then re‐expressed in transformed cancer cells, making it an attractive anticancer target.

### 3.1 Biological roles of MTHFD2

In the mitochondria, one‐carbon units are usually derived from serine and attached to a tetrahydrofolate (THF) molecule as methylene‐THF (CH_2_‐THF), further oxidised to formate and then shuttled to the cytoplasm, where formate can be used for *de novo* purine synthesis, thymidylate or methionine synthesis [74, 75]. In the cytosol, CH_2_‐THF oxidation is carried out in its entirety by the trifunctional (dehydrogenase‐cyclohydrolase‐synthetase) NADP‐dependent MTHFD1, while the bifunctional (dehydrogenase‐cyclohydrolase) NAD‐dependent MTHFD2L together with the monofunctional (synthetase) MTHFD1L is responsible for catalysing these reactions in the mitochondria. During early embryogenesis and in transformed cells, the mitochondrial dehydrogenase and cyclohydrolase activities are instead carried out by the MTHFD2 enzyme, suggesting an isoform switch from MTHFD2L to MTHFD2 during cancer transformation [76, 77, 78]. More recently, the MTHFD2 protein has been reported to also have a nuclear role, being colocated to the nucleus [79] and specifically at replication forks [80]. Perhaps in line with this, MTHFD2 was recently shown to have a noncatalytic role in promoting homologous recombination (HR) repair, through interaction between CDK1 and EXO1 [81]. Future studies should further interrogate the nuclear role of this enzyme.

### 3.2 MTHFD2 as a target for anticancer treatment

**Fig. 2:**
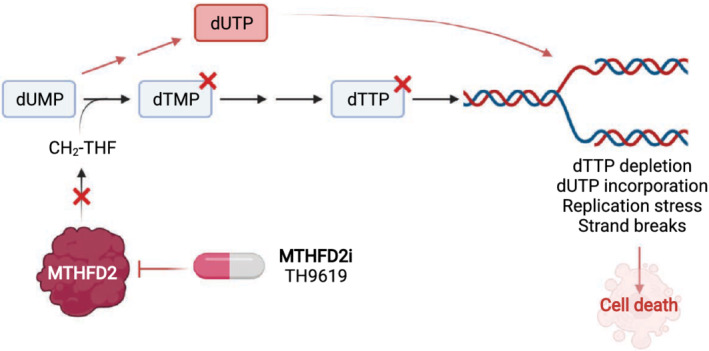
Mechanism of action of MTHFD2 inhibitors. Schematic detailing the proposed mechanism for antitumour effect of MTHFD2 inhibition. MTHFD2 supports de novo thymidylate (dTMP) synthesis by providing methyl‐tetrahydrofolate (CH_2_‐THF), and thus, loss of MTHFD2 activity depletes dTTP pools, leading to thymineless‐induced replication stress. In parallel, loss of dTMP production results in the accumulation of dUMP, the substrate of thymidylate synthase (TYMS), which is subsequently phosphorylated to its triphosphate form dUTP. Excess dUTP is incorporated into DNA leading to elevated genomic uracil exacerbating replication stress, ultimately resulting in DNA strand breaks and cell death. Figure created in BioRender. [Colour figure can be viewed at wileyonlinelibrary.com]

As MTHFD2 is one of the most upregulated metabolic enzymes in cancer [82], it has generated a lot of interest as a potential anticancer target. There are numerous reports supporting that MTHFD2 is required for survival in various cancers using RNAi approaches [83, 84, 85, 86, 87] or small molecule inhibitors [13, 88, 89, 90]. It is clear from the literature that MTHFD2 RNAi depletion is highly effective in killing most cancers. This appears to be related to MTHFD2 as expression of RNAi‐resistant MTHFD2 protein rescues the effect and mediates survival [13]. This is in sharp contrast to what is observed by MTHFD2 CRISPR‐Cas9 KO, where cancer cells survive by activating the serine hydroxymethyltransferase 1 (SHMT1) pathway [91]. The DepMap database [92] of CRISPR‐Cas9 KO cells supports that MTHFD2 is not required for cancer cell survival. There are mainly two inhibitor series to MTHFD2, the DS18561882 series [90] and the TH9619 series that we published [13]. In our hands, these inhibitors also inhibit MTHFD1 and the cell‐killing effect of these compounds could be related to targeting MTHFD1, rather than mitochondrial MTHFD2. The supporting information that TH9619 works by targeting MTHFD2 is as follows: (a) MTHFD2−/− cells are highly resistant to TH9619, while the same toxicity is observed in MTHFD1−/− cells, and (b) the toxic effects of TH9619 are reversed by metabolic rescue with thymidine, which also rescues cell killing by MTHFD2 siRNA, supporting the current model for mechanism of action (Fig. 2). However, since metabolic pathways are highly complex there could also be alternative explanations for these observed effects. Furthermore, the toxicity of MTHFD2 inhibitors is highly influenced by folate and other metabolite concentrations in the media [13]. One potential explanation for the effective killing with MTHFD2 RNAi but not with CRISPR‐Cas9 could be that cancer cells acutely rely on MTHFD2 to generate thymidine, but easily switch to use the SHMT1 pathway, allowing the clones to survive long term as in the case of CRISPR‐Cas9 KO cells. Interestingly, the MTHFD2 inhibitors are highly effective in killing a subset of cancer cells, but not all, indicating that neither the MTHFD2 RNAi nor CRISPR‐Cas9 KO cells predict the effects using the inhibitors. Edgetic‐like perturbation effects also appear relevant here and need to be considered when targeting MTHFD2.

## 4 SAMHD1

Targeting nucleotide metabolism is also important within the context of antimetabolite‐based cancer therapies, which typically target the DNA molecule via perturbation of both DNA and nucleotide metabolism [8]. This is exemplified with the enzyme SAMHD1, first described over two decades ago under the alternative name DCIP (dendritic cell‐derived IFN‐γ induced protein), owing to its identification as an orthologue of the mouse IFN‐γ induced gene *Mg11* from a human dendritic cell cDNA library [93]. DCIP was shown to be widely expressed by most human tissues and suggested to be a component of the innate immune response. The present name of SAMHD1, owing to a domain structure consisting of an N‐terminal SAM and central HD domain with conserved histidine (H) and aspartic acid (D) residues, was first referenced in 2009. Here, mutations in the *SAMHD1* gene were found to be responsible for a rare hereditary disorder called Aicardi–Goutières syndrome (AGS) [94], which is characterised by a defective innate immune response. However, the biological role(s) of SAMHD1 remained elusive. Subsequently, the dNTP triphosphohydrolase activity of SAMHD1 was characterised [95, 96], which also coincided with the identification of SAMHD1 as a human immunodeficiency virus type‐1 (HIV‐1) restriction factor in myeloid cells [97, 98]. Since then, our understanding of this enzyme has grown substantially, with additional biochemical activities and diverse biological roles reported, all of which paint a complex picture of the relationship of SAMHD1 with human health and disease, including cancer.

### 4.1 The biological roles of SAMHD1

SAMHD1 belongs to the HD‐domain superfamily, a group of metal‐dependent phosphohydrolases [99], and catalyses the hydrolysis of the α‐phosphate of dNTP molecules producing their cognate deoxynucleoside and inorganic triphosphate [95, 96]. The triphosphate moiety of a dNTP molecule is absolutely required by DNA polymerases for the DNA synthetic reaction. Thus, as the triphosphohydrolase activity of SAMHD1 removes the triphosphate moiety, this prevents the use of this dNTP molecule in DNA synthesis. The catalytic activity of SAMHD1 is regulated by nucleotide abundance [100, 101, 102, 103] (Box 1), and this elegant allosteric regulation mechanism is reminiscent of the key nucleotide metabolic enzyme and long‐standing anticancer target ribonucleotide reductase (RNR), which opposes SAMHD1 in the nucleotide metabolic scheme, being responsible for the reduction in nucleoside diphosphates (NDPs) to deoxynucleoside diphosphates (dNDPs). Similar to the broad substrate specificity of RNR, all canonical dNTPs (dGTP, dATP, dCTP, dTTP, and dUTP) can be accommodated in the catalytic site of SAMHD1 and subsequently hydrolysed [95, 96, 104], which is consistent with the notion that SAMHD1 is a major regulator of dNTP pools in human cells [105].

Box 1 **Allosteric regulation mechanism of SAMHD1**

• The catalytic activity of SAMHD1 is regulated by nucleotide abundance (reviewed in Ref. [100]);
• Catalytically active SAMHD1 is a homotetramer, and formation of this tetramer is dependent upon sequential nucleotide binding to distinct allosteric sites on each SAMHD1 monomer;
• Allosteric site 1 (AS1) binds specifically to guanine nucleotides, such as GTP or dGTP, which promotes the formation of the SAMHD1 dimer;
• Allosteric site 2 (AS2) binds to any dNTPs and then promotes dimerisation of these SAMHD1 dimers and thus formation of the catalytically active homotetramer [101, 102, 103].

In addition to the dNTP hydrolase activity of SAMHD1, several other activities of this enzyme have been documented. SAMHD1 was reported to have a nuclease activity [106, 107], in line with other HD‐domain superfamily members [99], which was tantalisingly consistent with other genetic defects known to cause AGS, as these were also nucleases (e.g. TREX1, RnaseH2). However, prior and subsequent biochemical studies indicated that SAMHD1 had no active site‐associated nuclease activity [96, 108, 109, 110, 111] and suggested the reported activity was likely a contamination in the preparation [109, 110]. Whether this activity exists and is biologically relevant remains in dispute [112, 113]. Interestingly, rather than possessing nuclease activity itself, SAMHD1 has since been shown to have a noncatalytic role in recruiting DNA repair nucleases to sites of DNA damage or stalled DNA synthesis [114, 115]. Also, it is this activity that substantially contributes to the role of SAMHD1 in suppressing the innate immune response [115], although studies implicate a role for the dNTP hydrolase activity also [116]. By recruiting DNA repair nucleases, such as MRE11, to stalled replication forks, SAMHD1 facilitates the processing of excess single‐stranded (ss)DNA that builds up at stalled forks and leads to IFN‐induction via cGAS‐STING when this ssDNA leaves the nucleus and enters the cytosol. Consequently, a lack of SAMHD1 has been reported to impede replication fork progression (independently of dNTP hydrolase activity) [115], and enhance the cytotoxicity of DNA damage‐inducing agents, such as IR and topoisomerase poisons, in addition to PARP inhibitors [114]. SAMHD1 has also been reported to suppress the innate immune response via direct interaction with NF‐κB [117]. Additionally, SAMHD1 possesses nucleic acid binding activity [108, 110], whose cellular role remains somewhat unclear but was recently shown to be important for antiretroviral activity [118].

### 4.2 Understanding the relationship between SAMHD1 and cancer

There is a strong link between the composition of dNTP pools and genome stability, and of course, this relationship is extremely important in cancer biology [119]. Similarly, the noncatalytic role of SAMHD1 in DNA repair and replication fork restart, together with its links to the innate immune response, also has important implications for our understanding of cancer [120]. Thus, perhaps unsurprisingly, dysregulation or mutation of SAMHD1 has been reported in several malignancies (reviewed in refs. [121, 122]). Chronic lymphocytic leukaemia (CLL) [123, 124], T‐cell prolymphocytic leukaemia [125], colon cancer [126] and mantle cell lymphoma [127, 128, 129], amongst others, have all had *SAMHD1* mutations identified within. Of course, without thorough characterisation of these mutants assessing their impact upon the various biochemical/biological activities of SAMHD1 (i.e. dNTP hydrolase, nuclease recruitment, innate immunity suppression, nucleic acid binding), it is difficult to hypothesise the outcome for cancer biology, given the impact of the loss of SAMHD1 can be hypothesised to have different outcomes depending upon the biological role in question. This approach was applied in a recent study characterising the colon cancer and leukaemia‐associated R366C/H mutant, and showed that while this mutation retains noncatalytic roles of SAMHD1, the dNTPase activity is abolished [130]. Accordingly, this mutation could contribute to elevated dNTP pools, which are commonly reported in cancer cells [131].

Exemplifying the potentially contrasting effects of loss of SAMHD1 and underscoring the need for systematic approaches to characterise SAMHD1 mutants, we can consider the example of replication fork progression. Impairment of the noncatalytic replication fork restart function of SAMHD1 could promote replication stress in cancer cells, a hallmark of this disease and known to be a double‐edged sword, capable of promoting tumour progression but also being a tumour suppressive mechanism. Conversely, loss of the dNTP catabolic activity of SAMHD1 would lead to expansion of dNTP pools, which could be anticipated to alleviate replication stress, consistent with the abundance of literature showing the rescue of replication stress in cultured cancer cells by treatment with exogenous nucleosides or their precursors [132, 133, 134, 135, 136]. However, replicating DNA with expanded dNTP pools, especially in the context of MMR deficiency, can result in elevated mutation rates [126], but several reports also note that a consequence of dNTP pool expansion can be cell cycle arrest at G1/S [105, 137]. This is an interesting observation that is consistent with findings reported in budding yeast using a constitutively active RNR mutant to expand dNTP pools [138]. Cell cycle arrest was attributed to perturbed assembly of preinitiation complexes at replication origins; whether this is the case in human cells with expanded dNTP pools remains to be investigated. Perhaps critically, these differential impacts of SAMHD1 upon cancer biology would be impacted by the cellular context, depending upon which oncogene is driving cancer cell proliferation [139], the metabolic wiring of the cell, and competency of genome stability pathways, for instance. Many open questions remain to be investigated here.

### 4.3 SAMHD1 is a drug resistance factor

It can perhaps be appreciated at this point that the relationship between SAMHD1 and cancer is somewhat complicated, and further investigation is required to delineate the different roles of this enzyme and their relevance to this disease. One instance in which there is a clear utility in targeting SAMHD1 in cancer is to improve the efficacy of a commonly used group of chemotherapies, antimetabolites, specifically nucleobase and nucleoside analogues [140]. As highlighted in the Introduction, these therapies were the proof of concept for the clinical utility of targeting nucleotide metabolism in cancer [6] and, accordingly, have been in clinical use for decades, being standard of care for many common malignancies.

These therapies are prodrugs and, owing to their similarity to endogenous nucleosides, are reliant upon the intracellular nucleotide biosynthetic and salvage machinery to generate their active phosphorylated metabolites, which are responsible for their anticancer effects. However, conversely, this renders these therapies subject to various nucleotide catabolic pathways that can also potentially inactivate them and reduce their efficacy, of which numerous examples exist, which we have discussed in detail previously [8]. Uniquely in human cells, SAMHD1 is a triphosphohydrolase and so can potentially convert active triphosphate metabolites of nucleoside analogues back to their respective inactive prodrug forms [140]. Studies probing the catalytic promiscuity of SAMHD1 began with the evaluation of nucleoside reverse transcriptase inhibitors (NRTIs) [141] and several base‐modified nucleotides [142] as SAMHD1 substrates. The first anticancer nucleoside analogue identified as a SAMHD1 substrate was the active metabolite of the antileukaemic drug clofarabine (Cl‐F‐ara‐ATP), which was also an AS2 activator [143]. Subsequent studies by ourselves and others confirmed and extended this finding to other anticancer nucleoside analogues, in particular, the deoxycytidine analogue cytarabine (ara‐C), which is standard‐of‐care therapy in acute myeloid leukaemia (AML) [14, 144, 145]. Here, the triphosphate metabolite (ara‐CTP) was shown to exclusively be a substrate of SAMHD1 [14, 144, 145], and accordingly, SAMHD1 could dictate the efficacy of this drug in a variety of preclinical AML models [14, 121, 144, 146]. Furthermore, establishing the clinical relevance of these findings, ara‐C treated AML patients with low SAMHD1 expression have a significantly better overall survival compared to those with high expression [14, 144, 147], clearly highlighting SAMHD1 as a therapeutic target in this context [140].

Subsequently, the active metabolites of many more anticancer nucleoside analogues have been identified as SAMHD1 substrates [148, 149, 150], and accordingly, SAMHD1 modulates the efficacy of some of these in disease models [14, 121, 144, 145, 146, 148, 149, 150, 151, 152, 153]. For the deoxycytidine analogue and DNMT1 inhibitor decitabine, clinically used in myelodysplastic syndrome (MDS) and AML, SAMHD1 expression also correlates with clinical outcome of patients receiving this therapy [148]. The deoxyguanosine analogue nelarabine, approved for use in refractory and relapsed T‐cell malignancies, is another interesting drug with regard to SAMHD1. The ablation of SAMHD1 expression sensitises cells to nelarabine (and ara‐G) [146, 151], and the lack of SAMHD1 expression in T‐cell acute lymphoblastic leukaemia (ALL) cell lines compared with B‐ALL cell lines explains the differential sensitivity observed [151]. Nelarabine is a rationally designed chemotherapy, based upon the observations that elevated dGTP is selectively toxic to T cells, which can occur through loss of purine nucleoside phosphorylase (PNP) (reviewed in ref. [154]). Notably, SAMHD1 has also been reported to protect cells from build‐up of cytotoxic dGTP, which could be exploited to target SAMHD1‐deficient cancer cells with PNP inhibitors [155, 156]. Thus, there is a striking parallel in the role of SAMHD1 in protecting cells from excess dGTP and the triphosphate metabolite of nelarabine (ara‐GTP). Given the apparent lack of SAMHD1 expression in T‐cell malignancy cell lines [151, 156], it is tempting to speculate that SAMHD1 (or rather lack of) is responsible for the original observations of dG and ara‐G selective T‐cell toxicity together with subsequent experiments, which formed the basis of nelarabine being developed as a T‐cell‐specific drug. Another interesting point is that many of the triphosphate metabolites of these nucleoside analogues can also allosterically activate SAMHD1 at the AS2 site [14, 143, 144, 145, 148, 149], but the biological relevance in cancer cells, if any, has been little explored/observed, perhaps owing to basal dNTPs already being sufficient for tetramerisation, which is known to be long‐lived [104].

### 4.4 Targeting SAMHD1 in cancer

**Fig. 3:**
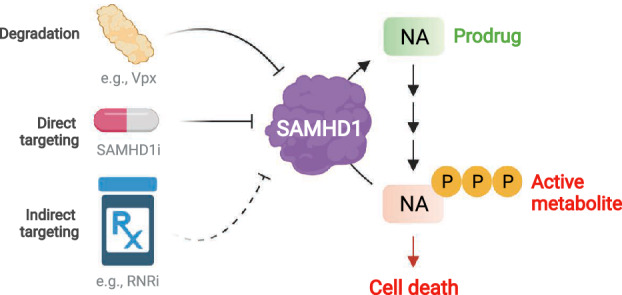
Strategies to target the drug resistance factor SAMHD1. SAMHD1 is a dNTP hydrolase that removes the triphosphate moiety from several anticancer nucleoside triphosphate analogues, thus converting the active metabolites of these therapies back to their inactive prodrug form. Current strategies to target SAMHD1 include proteasomal degradation, which can be achieved by delivery of the viral protein‐X (Vpx) into cancer cells. Direct catalytic inhibitors of SAMHD1 (SAMHD1i), which although have been documented against recombinant SAMHD1 *in vitro*, are yet to be shown to work in cell models. Indirect pharmacological approaches such as inhibitors of ribonucleotide reductase (RNRi) can suppress the drug resistance activity of SAMHD1 in cancer cells by perturbing nucleotide metabolism. Figure created in BioRender. [Colour figure can be viewed at wileyonlinelibrary.com]

Given the potential utility of inactivating SAMHD1 in cancer to enhance the efficacy of antimetabolites, coupled with potential applications in the immune response and viral infections, various approaches have been reported to target SAMHD1 (Fig. 3). We initially proposed the use of viral protein‐X (Vpx) as a biologic inhibitor of SAMHD1 to enhance ara‐C efficacy in AML which we demonstrated in cell models and primary patient material [14]. Vpx is a simian immunodeficiency virus (SIV) accessory protein that has evolved to target SAMHD1 for proteasomal degradation through interaction with the ubiquitin ligase DCAF1 [97, 98] essentially functioning as nature’s proteolysis‐targeting chimera (PROTAC). However, there are limitations to a protein‐based therapy, which we have discussed previously [146], and Vpx has additional cellular targets [157].

A direct pharmacological strategy, that is a SAMHD1 inhibitor, would be ideal and could also be an important tool for the scientific community to explore SAMHD1 biology and the consequences of dNTP pool expansion. Given the oligomeric nature of SAMHD1, together with distinct allosteric and catalytic sites, one would anticipate this is a druggable target. However, SAMHD1 inhibitors with demonstrated activity in cell models are yet to be reported. Several studies have documented high‐throughput amenable assays to screen chemical libraries [143, 158, 159, 160], and thus far, screening campaigns have focused upon libraries of FDA‐approved drugs [158, 159]. These studies have identified several chemotypes with micromolar inhibition against recombinant SAMHD1 protein *in vitro*, but little understanding of their inhibitory mechanism was provided together with no evaluation of their utility in cell models. In addition, one of these studies [158] also identified deoxyguanosine and its analogues, such as the antiviral acyclovir, as high micromolar SAMHD1 inhibitors *in vitro*, which could provide the future basis for fragment‐based chemical probe development efforts. Another approach to identify SAMHD1 inhibitors has stemmed from the use of nonhydrolysable dNTP analogues [143, 161, 162, 163]. Although these molecules have little use as chemical probes owing to their triphosphate moieties preventing cell permeability, plus the lack of selectivity offered by dN(TP) analogues, they have yielded great insights into the catalytic mechanism of SAMHD1 together with potential mechanisms of inhibition [161, 162, 163], which would inform future studies.

As a complementary approach to identify small molecule modulators, we recently reported a phenotypic screening strategy [15], which by default would yield cell‐active molecules. Here, we exploited the differential sensitivity of leukaemic cells to ara‐C depending upon SAMHD1 status [14] and screened libraries of molecules to identify those that can sensitise cells to ara‐C in a SAMHD1‐dependent manner. Although rather than a direct inhibitor of SAMHD1 dNTPase activity, our initial report from this screen illustrated the finding that the cellular ara‐CTPase activity of SAMHD1 can be suppressed indirectly with another class of anticancer drugs, RNR inhibitors (RNRi), which target the enzyme RNR responsible for the rate‐limiting step in *de novo* nucleotide biosynthesis. Although synergistic cell killing between various RNRi and ara‐C has been reported many decades ago, we found that in haematological cancer models and primary patient material, this synergy positively correlated with SAMHD1 protein abundance, and models lacking SAMHD1 displayed no synergy (which is the exact phenotype one would hope from a direct SAMHD1 inhibitor). Furthermore, RNRi could overcome SAMHD1‐mediated resistance to ara‐C in several mouse models of AML. Interestingly, the SAMHD1‐dependent sensitisation was observed only with nonallosteric inhibitors of RNR such as hydroxyurea (HU), gemcitabine (dF‐dC) and triapine (3‐AP), but not with allosteric RNRi exemplified by the purine analogues clofarabine, fludarabine and cladribine. Mechanistically, we proposed a model in which the changes in dNTP pools caused by nonallosteric RNRi treatment perturb the allosteric activation of SAMHD1 at AS2, which alters substrate specificity, specifically that dCTP‐activated SAMHD1 lacks ara‐CTPase activity [15]. Additional studies should interrogate this further, and also the wider applicability of this strategy, for instance to other nucleoside‐based drugs under SAMHD1 control in cancer cells.

This indirect pharmacological strategy, utilising already‐approved anticancer therapeutics, has several advantages over the use of yet to be developed direct SAMHD1 inhibitors. Critically, these findings can be rapidly translated into the clinic, especially as at least two nonallosteric RNRi (HU and dF‐dC) are currently employed in cancer treatment. As HU is already used in AML treatment, this has facilitated the establishment of a clinical study in Sweden to evaluate whether the addition of this drug can improve ara‐C standard‐of‐care therapy in newly diagnosed AML patients (EUDRACT: 2018‐004050‐16). Given we have shown that this combination did not affect the efficacy of anthracyclines in AML cell models [15], which is combined with ara‐C in AML standard of care, this strategy could also be further combined with attempts to refine anthracycline therapy [164]. Furthermore, RNRi has potent monotherapy anticancer activity that would not be expected from direct SAMHD1 inhibitors, which is an important consideration when designing optimal combination therapies to tackle heterogeneity within both patient and tumour populations [165]. This approach would also be expected to retain the other cellular roles of SAMHD1 relevant to human health (see Section 4.1). However, this indirect approach has limited utility in further understanding SAMHD1 biology that direct small molecule inhibitors would allow, and so further research efforts should establish these tools, which would allow comparison of these complementary targeting strategies.

## 5 Conclusions and future perspectives

The exploitation of the DDR, together with the differential DNA repair proficiency of cancer cells, holds much promise in the selective targeting of tumours, and there is much more exciting work to be done in this field. Here, we have discussed how this can be achieved through targeting nucleotide metabolic enzymes, which are involved in both the production and maintenance of dNTP pools in cancer cells. These same pathways also activate long‐standing anticancer drugs consistently used in cancer therapy. Thus, nucleotide metabolic enzymes, many of which are druggable, constitute encouraging anticancer targets, either to induce cytotoxic DNA lesions in cancer cells or to modulate the efficacy of existing cancer drugs. There are also other therapeutic uses of targeting nucleotide metabolism that have not been discussed here, such as its importance for cell differentiation, which can be exploited in AML treatment [166, 167], and the relevance for response to immune checkpoint inhibitors [168].

Targeting nucleotide metabolic pathways/enzymes, however, can be complicated. Noncatalytic roles of metabolic enzymes are becoming more and more apparent, exemplified by the role of SAMHD1 in replication fork restart or MTH1 binding of tubulin during mitosis. Although we would anticipate that a catalytic inhibitor would allow retention of nonenzymatic functions, there is evidence suggesting this is not always the case. Thus, a thorough characterisation of the various roles and activities of these enzymes needs to be carried out to fully understand the consequences of targeting them with small molecules. Also, while the use of CRISPR‐Cas9 dropout screens to identify genetic dependencies of cancer cell lines is a powerful tool to identify therapeutic targets, the use of stable CRISPR‐Cas9 KO cells to validate small molecules or targets also appears more complicated than initially thought. Metabolic pathways can be notoriously complex, and we would argue metabolic rewiring in nucleotide metabolism KO cell lines prevents overly simple interpretations of these experiments. Another point of caution is the targeting of isozymes, as is the case with MTHFD2. The development of selective inhibitors can be challenging, perhaps reminiscent of kinase inhibitor development, but various strategies can be employed to overcome this. This highlights the question of whether it is beneficial to inhibit all isozymes within a pathway, which could potentially reduce the risk of metabolic rewiring‐mediated resistance, or if selective inhibition of a single isozyme is preferential, only further experiments will tell and this will be context‐specific.

Here, we have discussed just two targets, which can be exploited to induce DNA damage in cancer cells, MTH1 and MTHFD2; however, there are many more. Nucleotide biosynthesis has been considered a nononcogene addiction of cancer cells, as dNTPs are required to fuel cancer cell proliferation, and accordingly, many of the enzymes in these pathways have been revisited time and time again in the context of cancer therapy [169]. However, given dNTP biosynthesis is unquestionably important for all dividing cells, caution should be applied when targeting potential pan‐essential genes [170]. Although it should be noted that the differential dependence of cancer versus noncancer cells upon *de novo* vs salvage nucleotide synthesis is an ongoing area of research, which could offer potential selective vulnerabilities of cancer cells. It is these same metabolic differences that could be responsible (at least in part) for the therapeutic windows observed with antimetabolites, being one of the reasons these therapies remain standard of care to this day. Nucleotide pool sanitation enzymes also constitute promising anticancer targets owing to the higher susceptibility of free bases within the dNTP pool to modification than their counterparts in DNA [171], which we have discussed in detail before [20]. Modified nucleotides can also originate from the DNA molecule, as is the case with epigenetic nucleotides, and enzymes involved in their subsequent metabolism can be exploited for cancer cell killing [172, 173].

In addition to the exploitation of new therapeutic targets, nucleotide metabolic enzymes are also critical in dictating the efficacy of antimetabolites. While in preclinical cancer research there is a clear focus on the development of new targeted therapies, traditional chemotherapeutics such as antimetabolites are still used daily and with high clinical impact, and this will likely be the case for many years to come. Despite decades of clinical use, there is still much left to be uncovered, as these therapies typically have complex and polypharmacologic mechanisms of actions, which could be another reason for their clinical success. Research efforts should focus on gaining a better understanding of how these drugs work, exploiting our knowledge of nucleotide metabolic enzymes and their links with the DDR and DNA repair, and develop strategies to refine their use. There are numerous examples of targeting dNTP metabolic enzymes to modulate the efficacy of these therapies [8, 20], and approaches such as the use of RNRi to indirectly target SAMHD1 are particularly interesting given this uses a cheap already existing cancer drug that can be redeployed to modulate chemotherapeutic efficacy, which can be particularly important when considering financial burdens associated with new therapies.

## Conflict of interest

TH is listed as an inventor on patents related to small molecule inhibitors of MTH1 and MTHFD2. TH and SGR have shares in Oxcia AB, which develops MTH1 inhibitors. TH has shares in One‐carbon Therapeutics AB, which develops MTHFD2 inhibitors.

## Author contributions

TH and SGR conceived and wrote the manuscript.
